# Presence of B Cells and Plasma Cells in Oral Lichen Planus

**DOI:** 10.30476/DENTJODS.2020.81804.0

**Published:** 2020-09

**Authors:** Nazanin Mahdavi, Pouyan Aminishakib, Nika Soltani

**Affiliations:** 1 Dept. of Oral and Maxillofacial Pathology, School of Dentistry, Tehran University of Medical Sciences, Tehran, Iran; 2 Postgraduate Student, Dept. of Endodontics, Faculty of Dentistry, Tehran Medical Science Islamic Azad University, Tehran, Iran

**Keywords:** B cell, Plasma cell, Oral lichen planus, Pathogenesis

## Abstract

**Statement of the Problem:**

Oral lichen planus (OLP) is a chronic inflammatory disease with unknown etiopathogenesis. It was believed that T cells played the major role in developing the lesions. It has been recently suggested that B lymphocyte cells (B cells) and plasma cells may play a role in OLP pathogenesis.

**Purpose:**

OLP is considered as a T-cell mediated disease. It was believed that the presence of B cells and plasma cells in the sub-epithelial inflammatory infiltrate, rules out the diagnosis of OLP. This study aims to investigate the presence of B cells and plasma cells in the inflammatory infiltrate of OLP. In addition, the association between the presence of B cells and plasma cells with histopathologic features of the lesion was assessed.

**Materials and Method:**

To assess the presence of B cells and plasma cells, 61 cases with the diagnosis of OLP were collected. The cases with definite clinical and histopathological diagnosis of lichen planus based on WHO criteria were included. For each case, demographic information and histological characteristics were recorded. Specimens underwent immunohistochemical (IHC) staining for CD20 and CD138 and the percentage of the positive cells were counted and scored.

**Results:**

CD20 positive cells existed in all OLP cases with the mean expression of 22.5%± 15.17% and small number of CD138 positive cells were seen in 62.3% of our cases with the mean expression of 4.74%±9.23%. No association was found between histopathological features and CD138 expression, however, CD20 expression level was higher in the cases with parakeratinized surface (*p*= 0.004).

**Conclusion:**

B cells existed in the inflammatory infiltrate of OLP in all cases. Small number of plasma cells could be occasionally found in OLP. Therefore, presence of B cells and plasma cells in the inflammatory infiltrate cannot rule out the diagnosis of OLP.

## Introduction

Lichen planus is a chronic inflammatory disease, which affects skin, nail, oral and genital mucosa [ 1 ]. Oral lichen planus (OLP) has a prevalence of 0.1% to 4% in the population with a female predilection [ 2 ]. OLP is usually found in buccal mucosa, tongue, and gingiva [ 3 ]. Different clinical types of OLP include reticular, plaque-like, atrophic, erosive, and bullous pattern with the reticular form as the most common. Some investigators suggest that the plaque-like and erosive lesions have a potential for malignant transformation and development of squamous cell carcinoma [ 4 , 7 ]. 

The histopathological features include hydropic degeneration of basal cell layer, sub-epithelial band-like infiltration of lymphocytes, parakeratinized epithelium, keratinocytes apoptosis, and focal hyperparakeratosis of the epithelium [ 8 ]. 

The etiology of OLP is not completely understood, however, lymphocytic infiltration supports the hypothesis that OLP is a cell-mediated immune reaction or autoimmune reaction to keratinocytes. The characteristics of band-like lymphocytic infiltration are found to be a clue to etiopathogenesis of OLP [ 6 , 8 , 9 ]. 

Oral mucosa is exposed to variety of allergic materials such as dental restorative and casting materials like dental amalgam and Nickel. Foods like cinnamon and drugs are known to induce allergic reaction in the oral mucosa namely oral lichenoid reaction (OLR). Although OLR lesions are indistinguishable from OLP both clinically and histopathologically, unlike OLP, they do not go under malignant transformation. Moreover, contrasting OLP, lichenoid reactions secondary to dental restorative materials, medications, or foods can be resolved by changing restorative material or drug or food habits. ‌Briefly, OLP and OLR are two distinct lesions with different causes that need different considerations [ 10 , 11 ]. Many studies have investigated the content of inflammatory infiltrate to distinguish OLR from OLP. Some researchers have attributed the presence of B-lymphocytes (B cell) and plasma cells in the lymphocytic infiltrate as one of the definitions of OLR lesions [ 12 , 14 ]. CD20 is a phosphor-protein expressed on B cells from pre B stage to the late stage of maturation. The expression decreases as B cells turn into plasma cells [ 15 ]. 

CD138 (syndecan-1) is a proteoglycan that facilitates cell-to-cell adhesion, cell and extra-cellular matrix interaction, cell differentiation and proliferation. CD138 is present on mature epithelial cells and plasma cells while is absent on endothelial and normal mesenchymal cells. Based on messenger RNA studies, CD138 is highly expressed on normal and neoplastic plasma cells and is absent on other cell types [ 16 , 18 ]. 

The aim of this study was to assess the presence of B cells and plasma cells in the inflammatory infiltrate of OLP by immunohistochemical (IHC) staining of CD20 and CD138 respectively. Moreover, the association between the presence of B cells and plasma cells with histopathologic features of the lesion was evaluated.

## Materials and Method

This study cross-sectional study was conducted in Oral and Maxillofacial Department, Dentistry Faculty, Tehran University of Medical Sciences. It was ethically approved by Ethics Committee of Tehran University of Medical Sciences (ethical code: IR.TUMS.VCR.REC.1-395.1036).

### Samples

The material in this study consisted of 61 biopsy specimens collected from the files of Oral and Maxillofacial Pathology Department, Tehran University of Medical Sciences from 2010 to 2017. The patients with the definite clinical and histopathological diagnosis of lichen planus with WHO criteria were included [ 19 ]. Patients with incomplete files, other autoimmune diseases including graft-versus-host disease (GVHD), lupus erythematous, history of medicine such as sulfonylurea, metformin, lorazepam and ketoconazole which are known to induce OLR [ 11 ], patients with single or unilateral lesions on buccal site and lesions that occurred in association with amalgam or glass ionomer tooth filings were excluded. Then the specimens, confirmed by an oral and maxillofacial pathologist, underwent IHC staining.

### Evaluation of Hematoxylin & Eosin (H&E) slides 

The sections were reviewed by an oral and maxillofacial pathologist to determine the histopathologic features as follows: keratosis, acanthosis, granulosis, spongiosis, hydropic degeneration of basal layer, lymphocyte exocytosis, epithelium separation, intensity of inflammation (mild, moderate, and severe) and the degree of epithelial dysplasia (no, mild, moderate, severe and SCC) [ 20 ]. 

### Immunohistochemistry

Formalin-fixed paraffin-embedded blocks were cut into 4µm-thick sections and left 24 hours on silicone-coated slides. Afterwards, the sections were deparaffinized in xylene and immersed in methanol with 3% hydrogen peroxide for 5minutes to eliminate endogenous peroxide activity. Subsequently, the specimens were washed by citrated and left in microwave for 5min for antigen retrieval, then were cooled in room temperature for 30 min and washed by tap water for 10min. sections were treated with phosphate buffer saline (PBS) for 5 min and non-serum protein for 30min consecutively. Monoclonal antibodies were applied as follows for each section: CD20 (B cell, clone L26, Dako, Copenhagen, Denmark) and CD138 (clone CD138, Dako, Copenhagen , Denmark) diluted 1:200 and 1:100 respectively. They were left for 1 hour in room temperature, and then treated with PBS. Diaminobenzidine solution (Vector, Burlingame, CA, USA) 0.3% was used to visualize reaction products. As the last step, the sections were counterstained with Mayer’s hematoxylin, dehydrated, and mounted. Normal tonsillar tissue was included as positive control for CD20. For CD138 internal control was used [ 9 ]. For negative control, sections were treated with normal saline and were confirmed to be unstained. Tonsillar tissue served as external control for CD20 and membranous staining of the oral epithelial cells served as the internal control for CD138.

### Scoring

For both markers, each section was examined by light microscope (OLYMPUS, BX51) at the magnitude of 200× in 10 randomly selected fields. The number of cells in the band-like inflammatory infiltration with membranous staining were counted and the percentage of stained cells were reported as grade 1 for no expression, grade 2 for less than 50% expression, and grade 3 for more than 50% expression [ 9 ]. 

### Statistical analysis

SPSS version 15.0 (SPSS Software Inc., CA, USA) was used to analyze the data. The correlation between CD20 and CD138 expression was assessed by Spearman correlation test. Fisher’s exact test and chi-square test were employed to assess the difference of CD20 and CD138 expression in sections with different pathological features. The p< 0.05 was considered statistically significant.

## Results

**Figure 1:**
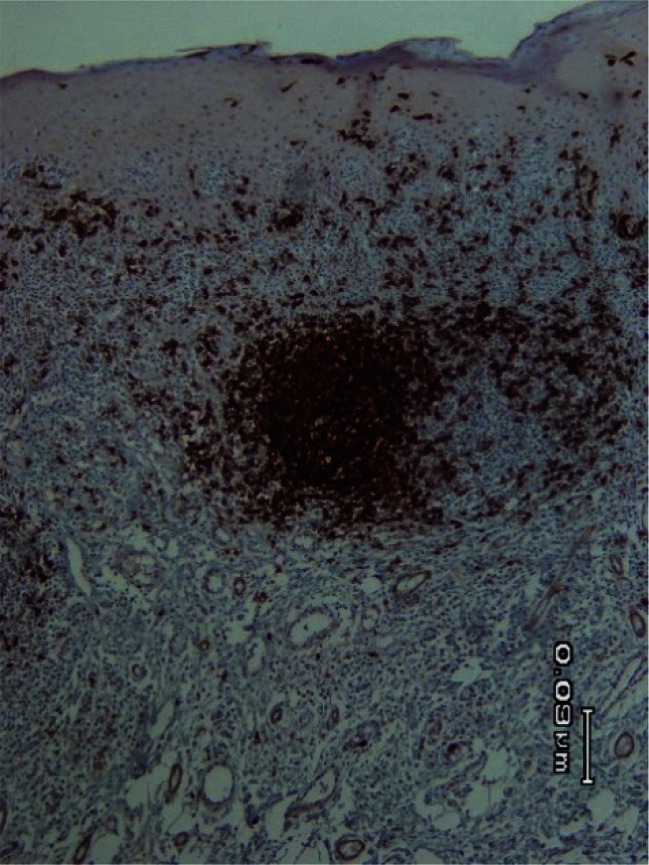
CD20 immuno-expression (×200).

**Figure 2:**
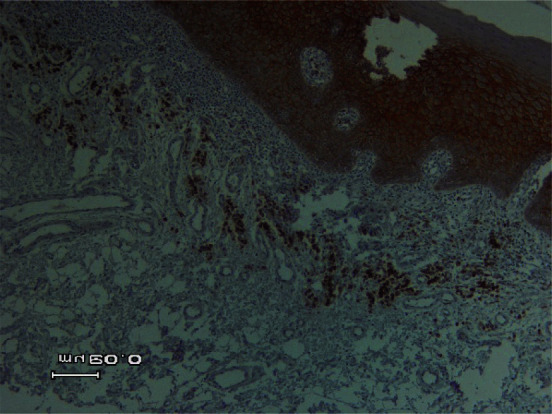
CD138 immuno-expression in lymphocytic infiltration and epithelium as internal positive control (200×).

Of the 61 specimens, 38(62.3%) belonged to female cases and 23(37.7%) belonged to male cases. The mean age of the patients was 49.6 years old. Buccal mucosa was the most frequent site of involvement (83.6%), and palate was the least frequent site with the frequency of 1.6 (Table 1). Epithelial dysplasia was found in 15 cases (24.6%). CD20 immuno-expression was found in all cases (Figure 1) while CD138 was expressed in 38 (62.3%) of cases (Figure 2). The mean expression of CD20 and CD138 were found to be 22.5%±15.17% and 4.74%±9.23%, respectively (Table 2). 

**Table 1:** Expression of CD20 and CD138 in OLPs (Oral lichen planus)

	Min (%)	Max (%)	Mean (%)	Expression by grade	
CD20 expression	2	80	22.5±15.17	Grade 1	0
				Grade 2	56(91.8%)
				Grade 3	5(8.2%)
CD138 expression	0	41	4.74±9.23	Grade 1	23(37.7%)
				Grade 2	38(62.3%)
				Grade 3	0

**Table 2:** Prevalence of clinical subtypes, histopathological features and their correlation with CD20 and CD138 expression.

	Number of specimens	Grade of CD138 staining intensity			*p* Value	Grade of CD20 staining intensity			*p* Value
		Grade 1	Grade 2	Grade 3		Grade 1	Grade 2	Grade 3	
Type of lesion									
Reticular	45(73.8%)	16(26.2%)	29(47.5%)	0	0.559	0	42(68.9%)	3(4.9)	0.522
Erosive/atrophic	13(21.3%)	5(8.2%)	8(13.1%)	0		0	11(18%)	2(3.3%)	
Bullous	3(4.9%)	2(3.3%)	1(1.6%)	0		0	3(4.9%)	0	
Site of lesion									
Buccal mucosa	51(83.6%)	17(27.9%)	31(55.7%)	0	0.345	0	46(75.4%)	5(8.2%)	0.785
Tongue	7(11.5%)	4(6.6%)	3(4.9%)	0		0	7(11.5%)	0	
Gum	2(3.3%)	1(1.6%)	1(1.6%)	0		0	2(3.3%)	0	
Palate	1(1.6%)	1(1.6%)	0	0		0	1(1.6%)	0	
Keratosis									
Parakeratosis	41(67.2%)	15(24.6%)	26(42.6%)	0	0.75	0	39(63.9%)	5(8.2%)	0.004*
Orthokeratosis	5(8.2%)	1(1.6%)	4(6.6%)	0		0	3(4.9%)	0	
Both	13(21.3%)	6(9.8%)	7(11.5%)	0		0	13(21.3%)	0	
No	2(3.3%)	1(1.6%)	1(1.6%)	0		0	1(1.6%)	0	
Acanthosis									
Yes	13(21.3%)	4(6.6%)	9(14.8%)	0	0.749	0	12(19.7%)	1(1.6%)	1.000
No	48(78.7%)	19(31.1%)	29(47.5%)	0		0	44(72.1%)	4(6.6%)	
Granulosis									
Yes	25(41%)	13(21.3%)	12(19.7%)	0	0.066	0	22(36.1%)	3(4.9%)	0.392
No	36(59%)	10(16.4%)	26(42.6%)	0		0	34(55.7%)	2(3.3%)	
Spongiosis									
Yes	8(13.1%)	2(3.3%)	6(9.8%)	0	0.698	0	7(11.5%)	1(1.6%)	0.518
No	53(86.9%)	21(34.4%)	32(52.5%)	0		0	49(80.3%)	4(6.6%)	
Lymphocyte exocytosis									
Yes	16(26.2%)	6(9.8%)	10(16.4%)	0	1.000	0	15(24.6%)	1(1.6%)	1.000
No	45(73.8%)	17(27.9%)	28(45.9%)	0		0	41(67.2%)	4(6.6%)	
Hydropic degeneration of basal layer									
Less than 25%	4(6.6%)	1(1.6%)	3(4.9%)	0	0.144	0	3(4.9%)	1(1.6%)	0.398
25%-50%	9(14.8%)	6(9.8%)	3(4.9%)	0		0	8(13.1%)	1(1.6%)	
More than 50%	48(78.7%)	16(26.2%)	32(52.5%)	0		0	45(73.8%)	3(4.9%)	
Epithelium separation									
Yes	22(36.1%)	7(11.5%)	15(24.6%)	0	0.586	0	19(31.1%)	3(4.9%)	0.341
No	39(63.9%)	16(26.2%)	23(37.7%)	0		0	37(60.7%)	2(3.3%)	
Intensity of inflammatory infiltrative									
Mild	40(65.6%)	16(26.2%)	24(39.3%)	0	0.108	0	37(60.7%)	3(4.9%)	0.843
Moderate	19(31.1%)	5(8.2%)	14(23.0%)	0		0	17(27.9%)	2(3.3%)	
Severe	2(3.3%)	2(3.3%)	0	0		0	2(3.3%)	0	
Dysplasia									
No	46(75.4%)	17(27.9%)	29(47.5%)	0	1.000	0	41(67.2%)	5(8.2%)	0.321
Mild	15(24.6%)	6(9.8%)	9(14.8%)	0		0	0	0	
Moderate	0	0	0	0		0	0	0	
Severe	0	0	0	0		0	0	0	
SCC	0	0	0	0		0	0	0	

No association was found between histopathological features and CD138 expression, however, CD20 expression level was higher in the cases with parakeratinized surface (*p*=0.004). The correlation between CD20 and CD138 was statistically significant (coefficient= 0.317 *p*= 0.013).

## Discussion

OLP is a chronic oral mucosal disease with autoimmune entity due to T cell lymphocytes predominance. The exact etiology and pathogenesis is unknown [ 21 ]. Many red and white lesions are developed in oral mucosa because of contact with restorative materials (like amalgam and glass ionomer), or foods (like cinnamon), and medications. Lesions like oral lichenoid drug reaction, oral lichenoid contact reaction, and GVHD are categorized as oral lichenoid lesions (OLL), which are indistinguishable from OLP clinically and histopathologically. Some articles have reported the presence of B cell and plasma cell in lymphocytic infiltrate but it was believed that presence of B cell and plasma cell was an indicative of OLR [ 10 , 12 ]. In this research, we confirmed the diagnosis of OLP based on clinical features, histopathological characteristics, and medical history. CD20 and CD 138 expression were assessed by IHC to evaluate the presence and intensity of B cells and plasma cells in OLP inflammatory infiltrate. In addition, the association of the presence of CD20 and CD138 with the histological features of OLP was assessed. There was no association between the histological features of OLP and expression of CD138 and the only association between histological features and CD20 expression was found in the pattern of keratosis; lesions with parakeratotic surface showed significant higher intensity of CD20 expression. In our study, B cells were present within the inflammatory infiltrate of all OLPs counting for less than 50% of the inflammatory cells in 91.8% of the cases. These findings are supportive of the hypothesis of the active role of B cells in the pathogenesis of OLP, which is consistent with Mattila et al. [ 9 ] that reported presence of B cells in 74.3% of OLP lesions as an indicative of B cells role in the pathogenesis of OLP. Plasma cells were found in 62.3% of the cases with the mean expression of 4.74%±9.23% that shows the presence of a small number of plasma cells in the inflammatory infiltrate of OLP does not rule out the diagnosis of OLP. These findings are in contrast with a number of previous studies, which reported B cell and plasma cell were uncommon in OLP [ 22 ]. Mravak-Stipetić et al. [ 23 ] showed that plasma cells were present in both OLP and OLL, which is consistent with the present study; however, the intensity of plasma cell was higher in OLL. 

CD138 presence can also develop a hypothesis about the role of plasma cell in the pathogenesis of OLP. Raybaud et al. [ 24 ] reported Epstein - Barr virus (EBV) infection in 74% of OLP with higher rate in erosive type and presence of higher number of plasma cells in OLP lesions infected with EBV. Nearly all EBV+ cells detected in OLP lesions were CD138+ plasma cells and more rarely CD20+ B cells, this means that plasma cells can play the role of host to EBV, and help the amplification of the virus particle. This finding is a newly discovered factor to OLP pathogenesis. We used CD20 to assess the presence of B cells in OLPs. CD20 is present on all maturation stages of B cell from pre B cell to immature and activated B cell [ 15 ]. There are some CD markers such as CD27 and CD5, which appear on special stages of maturation, and their assessment would be helpful in determining the type of B cell in lymphocytic infiltrate [ 9 ]. We suggest other future studies to evaluate the presence of EBV and other viral infections in sections to check their association with plasma cells. 

## Conclusion

B cells were found in all cases of OLP and few scattered plasma cells were seen in most cases. Presence of B cells and small number of plasma cells does not rule out the diagnosis of OLP.
